# In Situ Replacement of Infected Pseudoaneurysm of the Aortic Arch and Brachiocephalic Trunk Using Surgeon-Made BioIntegral Graft

**DOI:** 10.1155/crvm/8059936

**Published:** 2025-04-08

**Authors:** Joanna Halman, Łukasz Znaniecki, Piotr Siondalski

**Affiliations:** ^1^Department of Vascular Surgery, University Medical Center, Gdańsk, Poland; ^2^Department of Cardiac Surgery, University Medical Center, Gdańsk, Poland

## Abstract

An infection and aortic arch pseudoaneurysm can be fatal if not emergently and adequately treated. Optimal surgical procedures and optimal graft materials remain controversial. We describe a 61-year-old patient who underwent in situ repair of the infected pseudoaneurysm of the aortic arch. A porcine pericardium patch (BioIntegral Surgical Inc., Mississauga, ON, Canada) was used to reconstruct the aortic wall, followed by the reconstruction of the brachiocephalic trunk using a surgeon-made tube. The patient made a full recovery. Self-made tube grafts for in situ reconstruction offer many advantages and may be a valuable option.

## 1. Introduction

Mycotic aneurysms, caused mainly by bacterial infection, can form anywhere in the body [1, 2]. The initial clinical features of mycotic aneurysms, such as systemic sepsis, weight loss, or fever of unknown origin, can be misattributed to the source of infection, most commonly endocarditis. A mycotic aneurysm of the aorta can be fatal if not emergently and adequately treated [2, 3]. Bleeding and rupture usually occur in the bacteremic phase. Still, it is not uncommon to happen after the primary infection has been treated due to the erosion and weakening of the vessel wall. Optimal surgical procedures and optimal graft materials remain controversial [4, 5]. After treatment of infected aneurysms, recurrent infection is associated with high mortality and morbidity rates. We describe a 61-year-old patient who underwent in situ repair of the infected pseudoaneurysm of the aortic arch and brachiocephalic trunk.

## 2. Case Report

**Figure 1:**
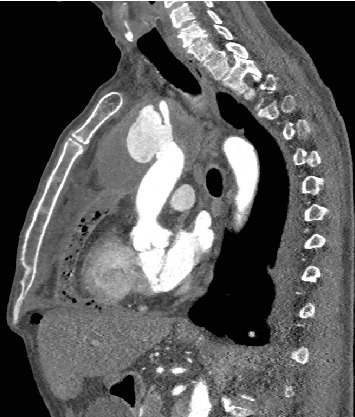
Sagittal view of the aortic arch on the angio CT scan illustrating pseudoaneurysm.

**Figure 2:**
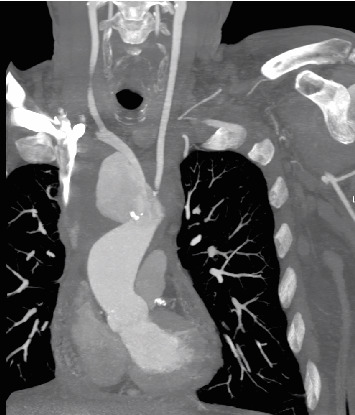
Coronal view of the aortic arch on the angio CT scan illustrating pseudoaneurysm.

**Figure 3:**
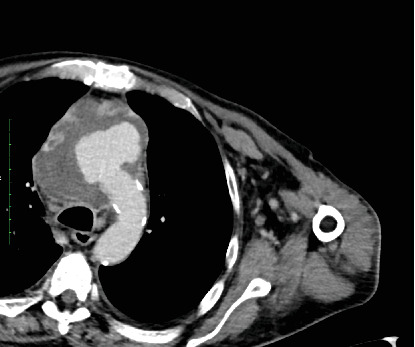
Axial view of the aortic arch on the angio CT scan illustrating pseudoaneurysm.

**Figure 4:**
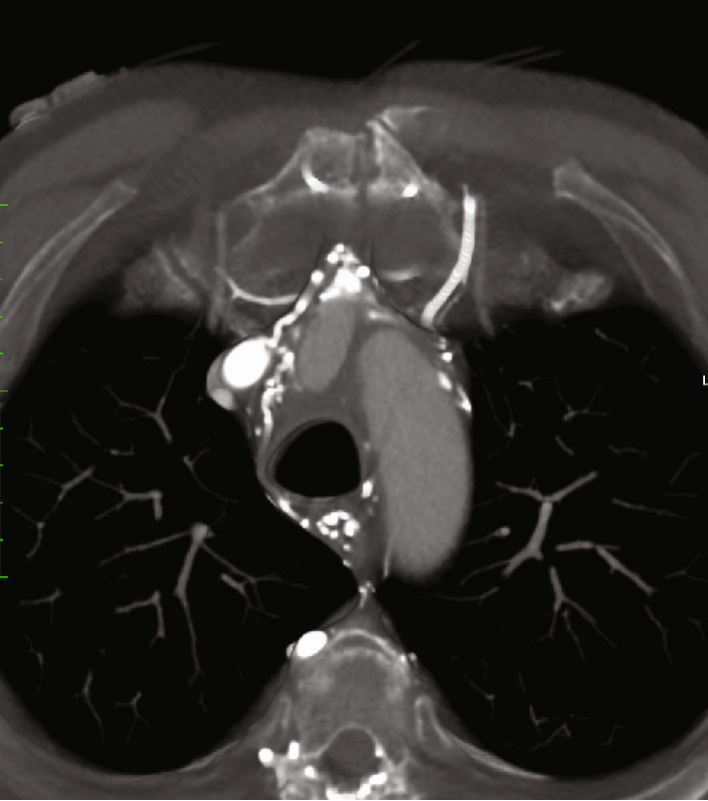
Axial view 10 months postoperatively.

**Figure 5:**
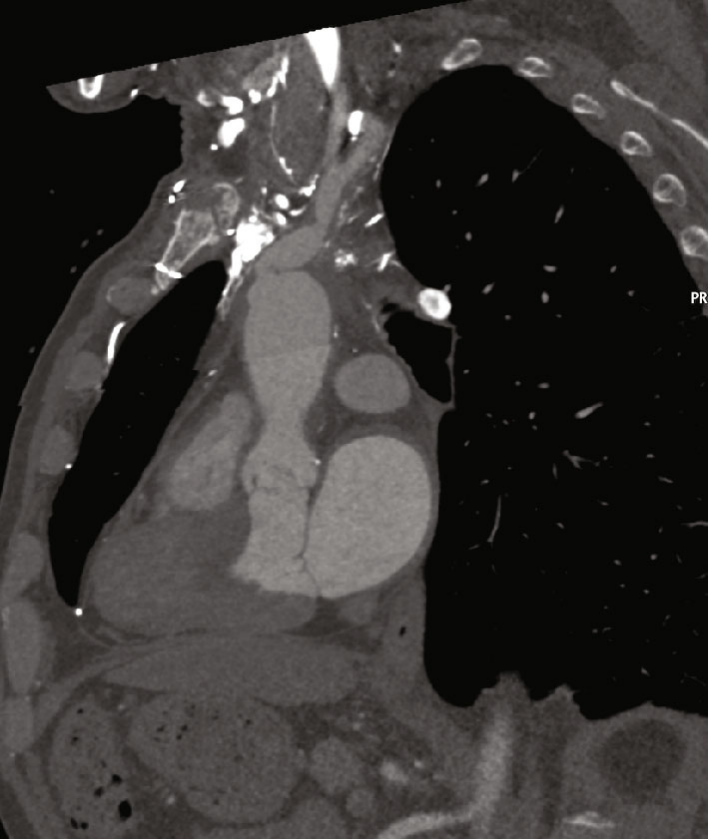
Sagittal view of the aortic arch 10 months postoperatively.

**Figure 6:**
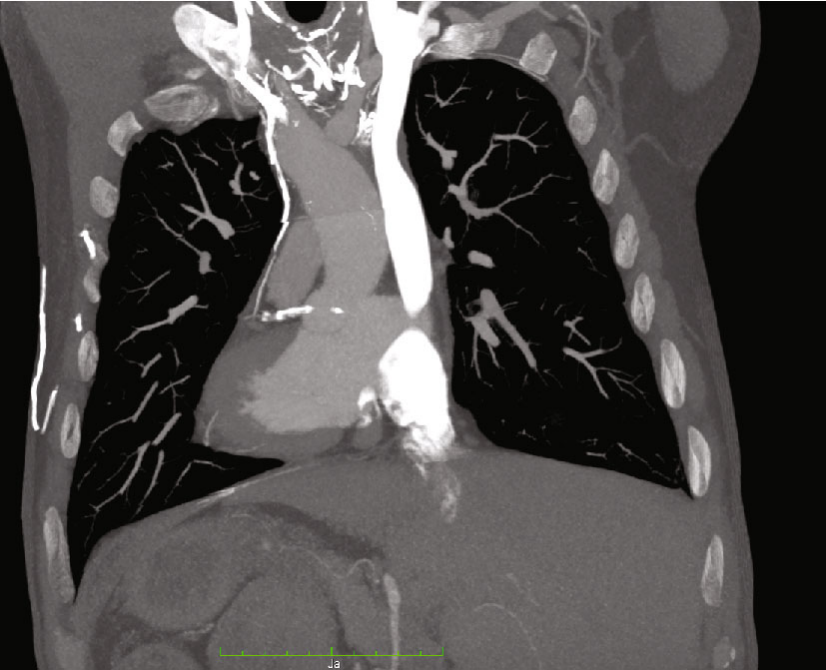
Coronal view of the aortic arch 10 months postoperatively.

A 61-year-old patient, previously treated for pericarditis and cardiac tamponade a week earlier, was readmitted due to chest pain and dyspnea. A CT scan performed at the emergency department revealed disruption of the ascending aortic wall, a pseudoaneurysm of the ascending aorta, and a brachiocephalic trunk (Figures 1, 2, and 3). At admission, the patient was stable and responsive. HgB level was 13.2 mg/dL, WBC 14.8 × 10^9^/L, and CRP 120 mg/L. Blood and pericardial fluid cultures revealed *Staphylococcus aureus*. The patient was qualified and prepared for the surgical repair of the aneurysm through ministernotomy. After the patient was systematically treated with heparin, cardiopulmonary bypass (CBP) was initiated via right femoral arterial and venous cannulation. Crystalloid cardioplegia was used. Intraoperative findings included a massive pseudoaneurysm over the aorta, superior vena cava, and right atrium. In addition, pericardial empyema was found. Pseudoaneurysm was opened longitudinally. The tear in the aortic arch and a massive tear almost entirely amputating the brachiocephalic trunk were identified. A porcine pericardium patch (BioIntegral Surgical Inc., Mississauga, ON, Canada) was used to reconstruct the aortic wall, followed by the reconstruction of the brachiocephalic trunk using a surgeon-made graft. The handmade roll was constructed by rolling up and stitching a rectangular patch. Debridement of remaining infected and necrotic tissues was performed. Irrigation of the thoracic cavity was performed. Total CPB time was 169 min. Drainage tubes were placed in the thoracic cavity and pericardium. During the postoperative course, the patient developed septic shock that was treated with intravenous cloxacillin and caspofungin for 16 days. The patient required multiple blood transfusions and dialysis. Sixteen days after the procedure, in stable general condition, he was transferred to the internal medicine ward for further treatment and rehabilitation. The patient made a full recovery. CT scan 10 months postprocedure showed no signs of infection or graft dilatation (Figures 4, 5, and 6).

## 3. Discussion

The majority of mycotic aneurysms are caused by bacterial infection. Among the most common pathogens are *S. aureus* and *Salmonella* sp. [2, 6]. The epidemiology of this disease is changing. Bacterial endocarditis, once the leading cause, now accounts for the minority of cases reported [1]. Mycotic aneurysms are relatively rare [2]. Symptoms are nonspecific and can include fever and uncontrolled systemic sepsis. However, the first symptom may manifest as complications of aneurysm expansion or rupture [6]. Strictly conservative management is associated with high mortality rates. Antibiotic therapy is recommended to be antibiogram-guided and implemented according to clinical progression. The definitive treatment remains surgical, even though perioperative mortality is high and, according to literature, reaches up to 63% in patients with aneurysm rupture [5, 6]. Even in nonretracture cases, aortic reconstruction in the infection setting is demanding. The choice of material is an important clinical decision. Currently, various options are available.

Autologous veins, considered favorable over prosthetic grafts, have the lowest reinfection rates of less than 2% but bear the most significant risk of rupture, aneurysm formation, thrombosis, and occlusion. Retrieving a vein prolongs the time of surgery, which is undesirable in unstable patients [7]. Other clinically accepted options are dacron grafts and rifampicin-soaked grafts, which are off-the-shelf solutions, but as a nonbiological material, they bear the most considerable risk of reinfection of all the available materials [8] with the reported occlusion rates between 5% and 9% [7]. The autologous pericardium has been reported to treat infective endocarditis, but the surface area is insufficient to reconstruct the great vessels. We decided to use a xenopericardial tube graft, which combines biological material advantages and an off-the-shelf solution. It is available in emergent settings, is simple to prepare, and is well-adapted for an aortic position. Czerny et al. reported excellent bovine pericardial tube graft results in treating prosthetic or endovascular graft infection in 15 patients [9]. Weiss et al. performed a retrospective analysis of 35 patients who underwent in situ aortic reconstruction using self-made bovine pericardial tube grafts, and even though with the reported 30-day mortality of 31%, early radiological and midterm clinical results were good [5]. Belkorissat et al. reported encouraging results for bovine pericardial tubes in aortic infection treatment in 12, with no graft-related deaths early or late complications [7].

## 4. Conclusion

Surgical treatment of aortic infection remains a high-risk procedure. Xenopericardial self-made tube grafts for in situ reconstruction are a promising option offering many advantages, allowing the definitive eradication of the infection. Despite satisfactory short and midterm results, periodic CT is needed to investigate long-term results in terms of the durability of the graft used for aortic reconstruction.

## Data Availability

All relevant data are available upon request.
